# Management of polyorchidism: Surgery or conservative management?

**DOI:** 10.4103/0974-1208.74166

**Published:** 2010

**Authors:** Ahmet Bayraktar, Erkan Olcucuoglu, Ilyas Sahin, Yesim Bayraktar, Sedat Tastemur, Emin Sirin

**Affiliations:** Department of Urology, Turkiye Yuksek Ihtisas Hospital, Ankara, Turkey; 1 Department of Urology, Hacettepe University School of Medicine, Ankara, Turkey; 2 Department of Anesthesiology and Reanimation, Numune Hospital, Ankara, Turkey

**Figure 1:**
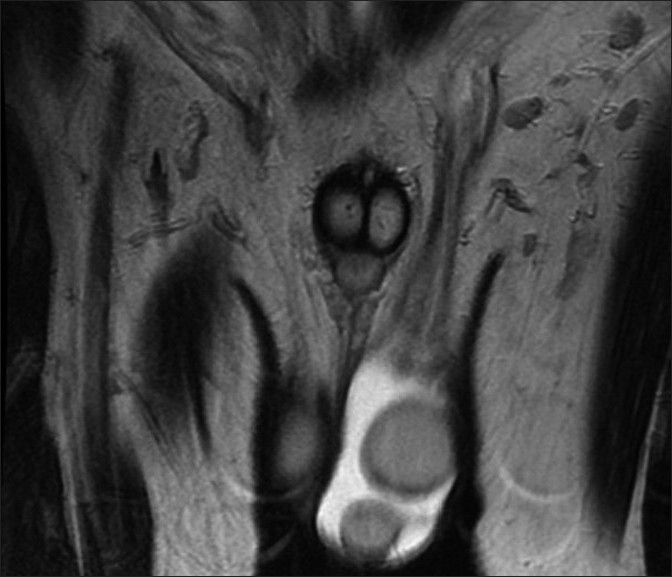
The presence of supernumerary testis that had the same signal characteristics as the normal testicles on MRI

Sir,

Polyorchidism is a rare congenital anomaly with more than 100 cases reported in the literature. We report a case of a 47-year-old man presented to our clinic (Department of Urology, Turkiye Yuksek Ihtisas Hospital, Ankara, Turkey) complaining of a painless mass in left hemiscrotum for 1 year. On admission, the patient denied any history of trauma, chronic illnesses, or major operations. The patient had no fertility problems and was a father of 3 children. On physical examination, a soft mass that is approximately the same size as left testis, and 2 vasa deferentia were noted in the left scrotum. The right testis and systemic examination were normal. The ultrasonography study of the left scrotum demonstrated a normal left testis measuring 28 × 34 × 36 mm and supernumerary testis measuring18 × 21 × 23 mm. The size and echogenicty of the right testis was normal. There were no suspicious malignant features on ultrasonography of the bilateral scrotum. The patient had normal serum levels of alfa fetoprotein, beta subunit of human chorionic gonadotrophin (hCG), lactate dehydrogenase, and testesterone. Further evaluation by magnetic resonance imaging (MRI) showed that the mass had intermediate signal intensity on T1-weighted image and high signal intensity on T2-weighted image. MRI confirmed the presence of supernumerary testis, which had the same signal characteristics as the normal testicles on T1- and T2-weighted images [Figure 1]. No other abnormal findings were observed. We did not perform surgical exploration on the basis of a combination of laboratory, ultrasonography, and MRI findings. During a 2-year follow-up, no clinical or radiologic progression was shown.

The most common presentation of polyorchidism is triorchidism, or tritestes, where 3 testes are present.[1] The supernumerary testis is most often located in the left side of the scrotum, as in this patient. Approximately 50% of the cases are in males between 15 and 25 years of age.[2] The most common anomalies associated with polyorchidism are maldescent (40%), hernia (30%), torsion (15%), hydrocele (9%), and malignancy (6%).[3] There are various classifications based on embryologic development, anatomical and functional arrangements of the testes and their outflow paths. We agree that the classification suggested by Bergholz *et al*., based on anatomy is a useful and more appropriate classification to use. A testis being drained by an outflow path (vas deferens) was coded as type A, whereas undrained testes without connection to a draining vas deferens were coded as type B. Type A testes were further divided into 4 subgroups. A1: Supernumerary testis has its own epididymis and vas deferens, A2: Supernumerary testis has its own epididymis but common vas deferens with neighboring testis, A3: Supernumerary testis has its common epididymis and vas deferens with neighboring testis, and A4: Supernumerary testis has its own vas deferens but common epididymis with neighboring testis. Type B testes were further divided into 2 subgroups. B1: Supernumerary testis has its own epididymis and B2: Supernumerary testis lacks an epididymis, thus consisting of testicular tissue only.[4] Using this classification, our case was type A1. Among the reported cases the most common type of polyorchidism was A3 (27%), followed by A1 (17%), and A2 (14%). Undrained testes (type B) counted for 18% of all described cases, of which 10% were type B1 and 8% B2.

There is no consensus in the literature regarding the management of polyorchidism, especially in cases where the supernumerary testes found incidentally are posing a surgical dilemma. Some authors have suggested orchiectomy of the supernumerary testes in adults because of 4%-7% risk of malignacy.[5,6] The low incidence of polyorchidism and its frequent association with proved risk factors, such as cryptorchidism, make it difficult to estimate its true malignant potential.[7] Although the appropriate management of polyorchidism remains unclear, there are some good ways to practice. If there is not any coexistent disorder, testicular tumor markers are negative for malignancy, and tumors can be ruled out by ultrasonography or MRI, and surgical exploration or biopsy is not necessary. Consequently, these patients can be followed-up conservatively.[8] The management of polyorchidism has been debated at length in the literature. On the basis of the literature, an intervention whether to use conservative treatment or surgery should be recommended based on the age of the patient, the reproductive potential, suspicion of malignancy, the position of the testis: scrotal or ectopic, association with cryptorchidism or torsion. In our view there is no need for surgical intervention in asymptomatic and normal testes. And close observation with physical examination, tumor markers, and imaging study (ultrasonography or MRI) in cases of polyorchidism is the proper management.
